# Lyophilization and homogenization of biological samples improves reproducibility and reduces standard deviation in molecular biology techniques

**DOI:** 10.1007/s00726-021-02994-w

**Published:** 2021-05-17

**Authors:** Agnes Molnar, Tamas Lakat, Adam Hosszu, Beata Szebeni, Anna Balogh, Laszlo Orfi, Attila J. Szabo, Andrea Fekete, Judit Hodrea

**Affiliations:** 1 SE Diabetes Research Group, Semmelweis University, Budapest, Hungary; 2 1st Department of Pediatrics, Semmelweis University, Budapest, Hungary; 3 ELKH-SE Pediatrics and Nephrology Research Group, Hungarian Academy of Sciences and Semmelweis University, Budapest, Hungary; 4 Department of Pharmaceutical Chemistry, Semmelweis University, Budapest, Hungary

**Keywords:** Lyophilization, Freeze drying, Tissue storage, Homogenization, Protein stability, RNA stability

## Abstract

Lyophilization is a cost-effective method for biological specimen preservation but detailed tissue-specific reference protocols are still lacking. Moreover, data are limited on the long-term stability of proteins and nucleic acids in lyophilized samples.

Here, we offer lyophilization protocols for various rat and mouse tissues (kidney, heart, liver, lung, aorta, and skin) coupled with technical hints for optimal sample preparation. We demonstrate that lyophilized samples stored at 4 °C for 20 months can yield protein and RNA of similar quantity and quality to −80 °C storage, while phosphorylated proteins are preserved as well. Freeze-dried and subsequently pulverized samples can provide more consistent, more reliable data especially when investigating focal injuries, such as fibrosis. We developed a protocol for the concentration of biological solutions and achieved 20-times concentration in human peritoneal dialysis effluent solution which enables the previously unattainable detection of proteins in these samples. We established a method for water removal as well as accurate water content measurement of fecal samples, which can be valuable for gut metabolome analysis.

Taken together, lyophilization is a valuable tool for the preservation of biological samples with many advantages. We aim to draw attention to the wide range of possibilities offered by freeze drying in pre-clinical or basic research.

**Supplementary Information:**

The online version contains supplementary material available at 10.1007/s00726-021-02994-w.

## Introduction

**Fig. 1:**
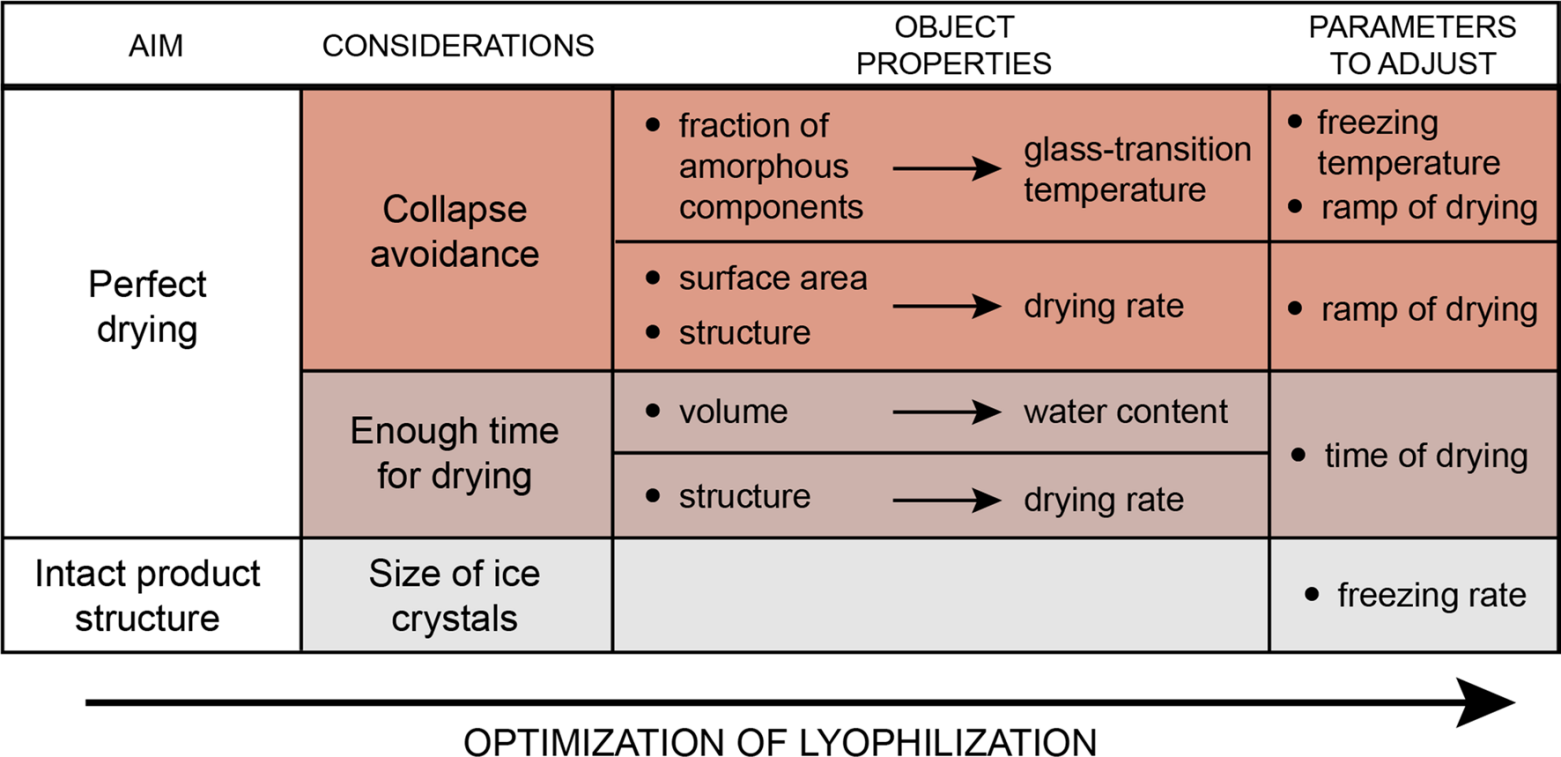
Modifiable process variables and crucial factors to consider for lyophilization optimization. To achieve perfect drying and to keep product structure intact, we need to avoid sample collapse, provide enough time for total drying and control the size of forming ice crystals. Ramp and time of drying, freezing rate, and temperature need to be adjusted based on the specific properties of different samples such as volume, structure, composition, and surface area. Exact physical properties of specific samples such as water content, drying rate, and glass transition temperature are usually determined by meticulous analytical measurements for cycle-optimized professional industrial usage. However, running a longer, safety optimized protocol based on estimated values is sufficient for laboratory application

Water removal by vacuum freeze-drying of biological materials is a first-rate method of preservation that yields final products of the highest quality. Pharmaceutical applications include drying of labile pharmaceutical products, production of long-term stable vaccine formulations, preservation of nucleic acid-based pharmaceuticals, and development of micro- and nanoparticulate systems for drug delivery (Kasper et al. 2013). Despite the numerous advantages, freeze drying has not become a routine method in research laboratories yet. Specific lyophilization protocols are scarce in the literature; the available recommendations lack specific details and are mainly limited to industrial use or a few specialized research applications (Chen et al. 2010; Fonte et al. 2016; Fornaguera et al. 2019). Thus, fine-tuning the right setups is labor-intensive and time-consuming for the everyday researcher (Tsinontides et al. 2004). Previous research demonstrated by quantity and quality measurements as well as biomolecular research methods such as RT-qPCR, copy number variation analysis, Western blot, gelatin zymography, SDS–PAGE, or even enzyme activity analysis that nucleic acids and proteins are well preserved in lyophilized samples (Damsteegt et al. 2016; Mareninov et al. 2013; Wu et al. 2012), however, the maximum storage time that has been investigated was 1 year. The technique not only offers an appealing, yet untapped potential for long-term storage, but the use of lyophilized tissue samples may also improve validity and reproducibility and may reduce scatter in molecular biology research. Upon new protocol assessment one has to consider several process variables that influence the freeze-drying cycle (Tang and Pikal 2004): freezing and drying times, temperature ramps, and pressure levels should be adjusted individually depending on product composition, structure, and volume (Fig. 1).

Beyond the biological advantages lyophilization may also facilitate sustainability. In today's highly collaborative scientific community there is a growing need to transport biological samples. Liquid nitrogen or dry ice is not required for the storage of freeze-dried samples, which significantly reduces shipping and storage costs (Lewis et al. 2016). However, data are limited on whether RNAs and proteins, as well as post-translational modifications such as phosphorylation are preserved in freeze-dried samples stored for long periods of time (20 months or more).

**Fig. 2:**
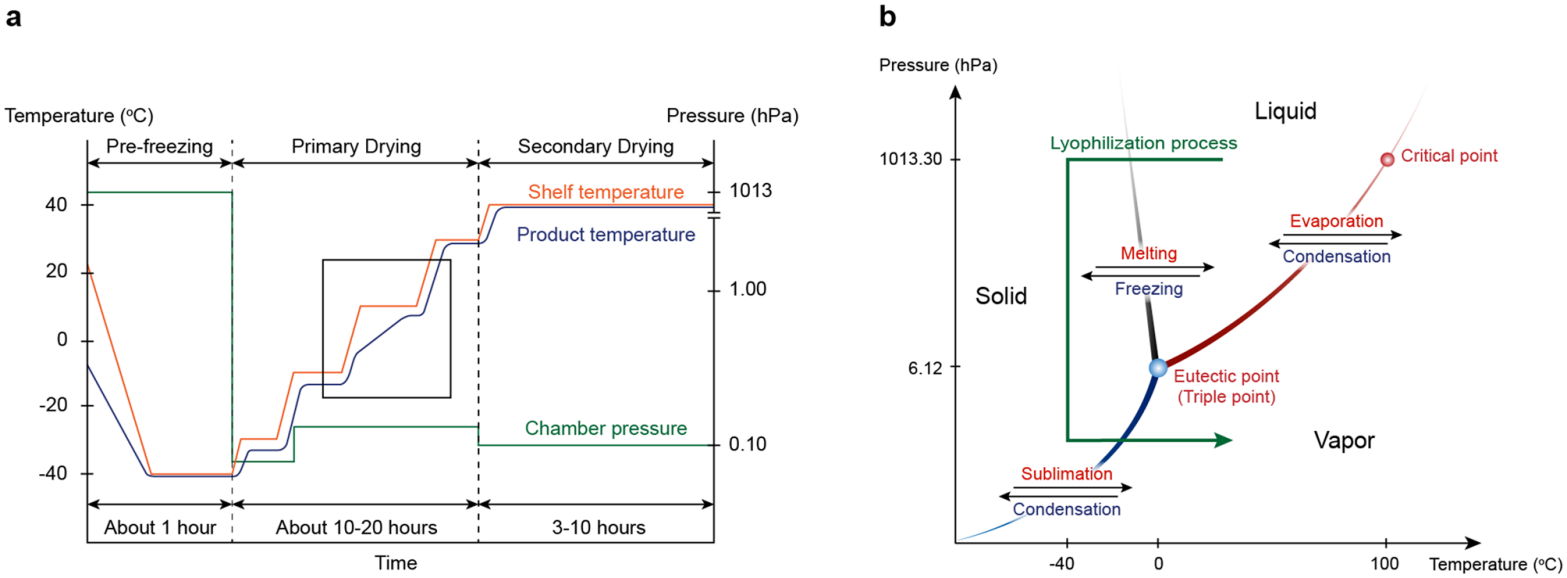
Theoretic background of lyophilization. **a** Typical freeze-drying cycle: About 1 h pre-freeze ensures the product is below its eutectic temperature to avoid collapsing before chamber pressure is lowered. Shelf temperature is gradually increased until all the ice is removed from the product at the end of 10–20 h of primary drying. During sublimation the product temperature increment slows down (black framed area), which can be monitored by an appropriate temperature sensor placed in the sample. Chamber pressure should be below vapor pressure at all temperatures, but too low pressure makes sublimation slower due to lack of driving force. Eventually, 3–10 h-long secondary drying is applied at lower pressure and higher temperature to remove sorbed water and moisture. **b** Phase diagram of water with the lyophilization process indicated: product is frozen under normal atmospheric conditions, then at a temperature below the triple point pressure is lowered to reach vapor pressure where ice sublimates

Lyophilization is a complex process consisting of three main steps: pre-freezing, primary drying, and secondary drying (Fig. 2a). Sublimation is the principle driving force of primary drying (Fig. 2b), which facilitates solvent removal from the sample, while product structure remains intact and enzymes stay inactive. For cycle optimization industrial application requires precise determination of the primary-drying endpoint, which is the longest part of the process. There are several methods for determining the primary-drying endpoint (Pisano 2020) however, most of these require analytical instruments that are not standard equipment in research laboratories. In the case of occasional use reducing process time usually takes a back seat behind elegance of the product and final moisture content. Therefore, our experiments were performed with a trial-and-error approach. According to this, our main consideration during the setup was to establish a protocol which results in a properly pulverizable product without any visible sign of residual moisture and the alteration of the structure. Images of lyophilized products can be found in Supplementary Figure 3. Furthermore, we used a Pt 100 probe to monitor ice sublimation. However, this method indicates the end of sublimation only in the region of the sensor, thus prolonged primary-drying time is recommended to avoid incomplete drying (Pisano 2020). In addition, lyophilized products were tested by further biological measurements to confirm the procedure’s efficiency. Finally, a shorter final section of secondary drying with higher temperature enables molecularly absorbed moisture to evaporate as well (Pisano et al. 2012). Based on the hypothesis that lower pressure improves drying rate, pressure is often decreased further during secondary drying. This is a general practice that we also applied. Duration of the pre-freeze step depends on initial sample temperature and volume. Primary drying takes about 10–20 h, while secondary drying is completed in 3–10 h depending on sample properties.

**Fig. 3:**
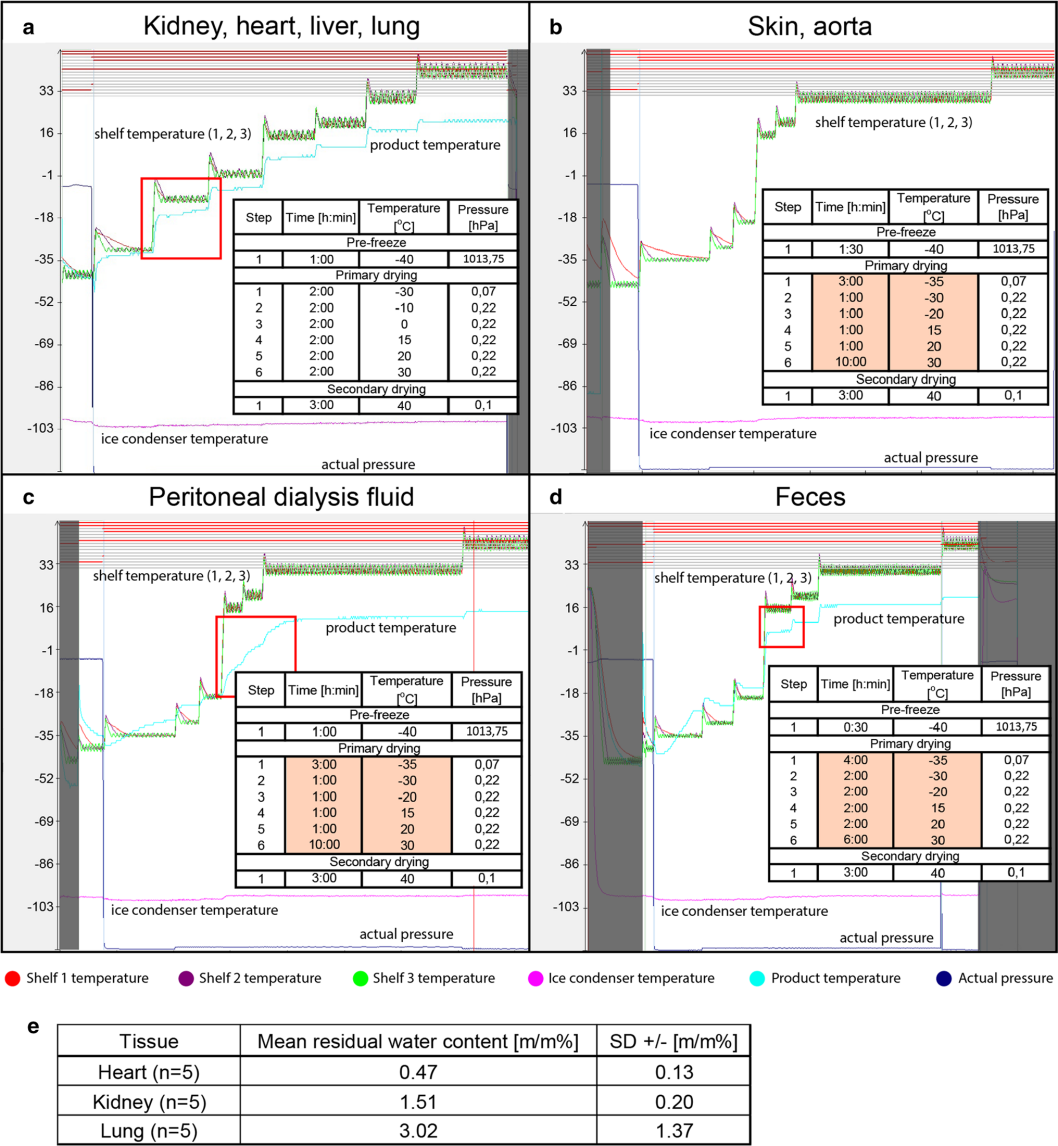
Protocols for lyophilization and their efficacy for various samples. Representative graphs and reference protocols with exact time, temperature, and pressure values indicated for each phase used for: **a** kidney, heart, liver, and lung tissue samples; **b** aorta and skin tissue samples; **c** peritoneal dialysis fluid concentration; **d** liquid removal from fecal samples. Red frames on representative graphs indicate the ice sublimation around the sensor when measured product temperature approaches shelf temperature. **e** Result of residual water content measurement of some limited tissue samples by Karl Fischer titration

This paper offers detailed protocols for rat and mouse kidney, heart, liver, lung, and skin tissue lyophilization, human peritoneal dialysis (PD) fluid concentration, as well as water and solvent removal from human fecal samples. We analyzed whether freeze-dried tissue samples can be stored at 4 °C for 20 months or more without quality impairment of RNAs and proteins. We conducted experiments to investigate the hypothesis that lyophilization and subsequent homogenization can be a superior method for sample preparation when investigating focal organ injuries, specific protein identification in human PD effluent (PDE) and metabolome analysis of fecal matter. Lyophilization may be an ideal choice in unexpected events such as the present pandemic when transportation and storage of biological materials at −80 °C is obstructed.

We hope to support the scientific community to spare time, effort and money, while drawing attention to the wide range of possibilities offered by freeze drying in pre-clinical or basic research.

## Materials and methods

### Animal experimental protocol

8-week old male Wistar rats (weighing 180–200 g) were purchased from “Toxi-Coop” Toxicological Research Center (Dunakeszi, Hungary). Animals were kept under standard laboratory conditions (in plastic cages under 12-h dark/light cycle at constant room temperature) with free access to standard rodent chow and drinking water. Diabetes was induced by a single intraperitoneal injection of streptozotocin (65 mg x bwkg^−1^) dissolved in 0.1 M citrate buffer (pH 4.5). 72 h later blood glucose levels were measured from tail vein with a D-Cont IDEAL device (77 Elektronika, Budapest, Hungary) after overnight fasting. Animals with ≥ 15 mmol/L peripheral blood glucose concentrations were considered diabetic. Age-matched non-diabetic animals injected with equivalent volumes of citrate buffer served as controls. At the end of the 7-week experimental protocol anaesthetized rats (intraperitoneal mixture of 75 mg × bwkg^−1^ Ketamine (Richter Gedeon, Budapest, Hungary) and 10 mg × bwkg^−1^ Xylazine (Medicus Partner, Biatorbagy, Hungary) were sacrificed by terminal blood draw. Kidney, heart, liver, lung, abdominal aorta, and furless skin samples were collected and immediately snap-frozen for further investigation.

### Human sample collection

PDE samples were collected from children receiving PD treatment at the 1^st^ Department of Pediatrics, Semmelweis University. All patients were dialysed with PD solution containing 1.5% glucose (Fresenius Medical Care, Bad Homburg v.d. Höhe, Germany) and all specimens were taken at the first dialysis of the morning after overnight home dialysis. Samples were immediately frozen at −80 °C until further processing.

Fecal samples were collected from healthy, inflammatory bowel disease, and irritable bowel syndrome patients at the 1st Department of Pediatrics, Semmelweis University. Specimens were kept refrigerated up to 24 h, then aliquoted and kept frozen at −80 °C until further use.

### Extraction for metabolome analysis

Human fecal samples were prepared for metabolomics analysis as described in Application Note 35,035 for MxP® Quant 500 kit of Bioctares (Innsbruck, Austria) (Heischmann 2018). To test the applicability and efficiency of lyophilization a custom extraction was performed as follows; two portions of 20–60 mg wet feces were processed and first, the wet specimen was lyophilized. The mass of the dry specimen was subsequently measured and recorded. Extraction of the first fraction was performed using ethanol-20 mmol/L phosphate buffer (pH = 7.5) 85:15 (v/v), while extraction of the second fraction was performed using a 15:85 (v/v) composition of the same liquids. 500 µL mixture was added to the dry sample with the container immersed in an ice bath and was shaken on an orbital shaker for 30 min at 200 rpm. The extraction procedure was repeated twice using each mixture, and the extracts were combined for metabolomic analysis.

### Lyophilization and product processing

Freeze drying and setup of various protocols was performed with a ScanVac CoolSafe Touch Superior device (LaboGene A/S, Allerod, Denmark). Collected tissue samples were cut in tiny (~ 20 mm^3^) pieces and stored at −80 °C optimally arranged in 2 mL plastic tubes to attain the largest surface possible. Tubes remained open throughout the entire process. Sublimation of the samples was monitored using a Pt 100 temperature sensor placed in the core of a chosen piece of tissue. Dried tissue products were manually smashed with 20 Gauge needles and pulverized with 5 mm stainless steel balls using a TissueLyser LT (Qiagen GmbH, Hilden, Germany) device. PD effluent fluid was snap-frozen on the wall of the container by spinning in liquid nitrogen to increase drying surface. Powdered tissue samples were stored at 4 °C for 20 months and then Western blot and RT-qPCR measurements were performed to compare the protein and RNA stability in lyophilized and conventional frozen samples. For detailed protocol including images of the samples please see Supplementary material. Residual water determination was performed using the well-known amperometric Karl Fischer titration, which is based on the measurement of current increase at constant voltage (Water: Semi-micro determiantion 2018).

### Reverse transcription quantitative polymerase chain reaction (RT-qPCR)

Total RNA was isolated from frozen- and from lyophilized homogenates of the same kidney and heart samples. Extraction was performed with Total RNA Isolation Mini Kit (Geneaid Biotech, New Taipei City, Taiwan) according to the manufacturer’s protocol. Quality and concentration of total RNA were measured on a NanoDrop ND-1000 spectrophotometer (BCM, Huston, TX, USA). 250 ng of first strand cDNA was reverse transcribed using Maxima First Strand cDNA Synthesis Kit for RT-qPCR (Thermo Fisher Scientific, Waltham, MA, USA). RT-qPCR was performed on a LightCycler 96 system (Roche Diagnostics, Mannheim, Germany) to determine specific mRNA expressions. Reaction mix contained 1 μL cDNA, 10 μL SYBR Green I Master enzyme mix (Roche Diagnostics, Mannheim, Germany) and 10 pmol × μL^−1^ of each specific primer (IDT, Coralville, IA, USA) designed based on nucleotide sequences from the National Center for Biotechnology Information’s nucleotide database (primer sequences: Table 1). Qualitative and quantitative analysis were performed by LightCycler 96 software version 1.1.0.1320 (Roche Diagnostics, Mannheim, Germany). All data quantification was normalized to Rn18s housekeeping gene expression from the same samples.

**Table 1:** RT-PCR primer sequences used in this study (GAPDH: Glyceraldehyde 3-phosphate dehydrogenase; T_a_: annealing temperature)

Gene name	Regular name	NCBI ID	Primer pairs		Product length	*T*_a_
*Gapdh*	Rat GAPDH	NM_017008.4	Forward:	5′-CAC CAC CAT GGA GAA GGC TG-3'	240 bp	60 °C
			Reverse:	5′-GTG ATG GCA TGG ACT GTG-3'		
*Acta2*	Rat alpha smooth muscle actin	NM_031004.2	Forward:	5′-GAG CGT GGC TAT TCC TTC GTG-3'	106 bp	60 °C
			Reverse:	5′-CAG TGG CCA TCT CAT TTT CAA AGT-3'		
*Rn18s*	Rat 18S ribosomal RNA	NR_046237.1	Forward:	5′-GCG GTC GGC GTC CCC CAA CTT CTT-3'	105 bp	60 °C
			Reverse:	5′-GCG CGT GCA GCC CCG GAC ATC TA-3'		

### Western blot analysis

Total protein was extracted from frozen- and from lyophilized homogenates of the same kidney and heart samples. Lysis buffer (1 M Tris, 0.5 M EGTA, 1% Triton X-100, 0.25 M NaF, 0.5 M phenylmethylsulfonyl fluoride, 0.5 M sodium orthovanadate, 5 mg × mL^−1^ leupeptin, and 1.7 mg × mL^−1^ aprotinin, pH 7.4) was used to homogenize samples and lysates were centrifuged at 13,000 rpm, 4 °C for 10 min. Supernatant protein concentrations were measured by detergent-compatible Bradford dye-binding method protein assay kit (Bio-Rad Hungary, Budapest, Hungary). Rehydrated PD effluent was used as total protein solution without preprocessing. 20 µg of denatured proteins were electrophoretically separated on 4–20% gradient Mini-PROTEAN TGX SDS–polyacrylamide precast gel (Bio-Rad Hungary, Budapest, Hungary) and transferred to nitrocellulose membranes. Membranes were blocked in 5% w/v non-fat dried milk in Tris-buffered saline (TBS) for 1 h at room temperature and probed with specific primary antibodies overnight at 4 °C, followed by horseradish peroxidase-conjugated secondary antibodies (Cell Signaling Technology, Leiden, Netherlands) (Table 2). Chemiluminescence detection was achieved with Molecular Imager VersaDoc MP 4000 System (Bio-Rad Laboratories, Hercules, CA, USA) using Luminata Forte (Millipore Corporation, Billerica, MA, USA) substrate. Bands of interest were analyzed using Quantity One Analysis 4.6.6 software (Bio-Rad Laboratories, Hercules, CA, USA) based on densitometry. Following background subtraction bands were normalized for Ponceau S staining to eliminate bias due to variations in total protein loading.

**Table 2:** List of antibodies used in this study for Western blot (αSMA: alpha smooth muscle actin; pAkt: phospho-protein-kinase B; peNOS: phosphorylated endothelial nitric-oxide synthase, CTGF: connective tissue growth factor)

Antibody	Vendor	Catalogue number	Dilution	Secondary antibody	Secondary antibody dilution	Secondary incubation time
Anti-αSMA	Sigma–Aldrich, Darmstadt, Germany	A2547	1:500	Goat anti-mouse IgG	1:6000	30 min
pAkt	Cell Signaling Technology, Leiden, Netherlands	4060S	1:1000	Goat anti-rabbit IgG	1:4000	30 min
Akt	Cell Signaling Technology, Leiden, Netherlands	9272S	1:1000	Goat anti-rabbit IgG	1:3000	1 h
peNOS	Cell Signaling Technology, Leiden, Netherlands	9571S	1:1000	Goat anti-rabbit IgG	1:3000	1 h
eNOS	Abcam, Cambridge, UK	66,127	1:1000	Goat anti-rabbit IgG	1:5000	1 h
CTGF	Santa Cruz Biotechnology, Dallas, USA	sc-14939	1:1000	Rabbit anti-goat IgG	1:5000	1 h

### Renal histology

Rat kidney tissues were fixed in 4% formaldehyde and embedded in paraffin. Masson’s trichrome stain was applied to 5 µm thick sections. Specifically stained fibrotic interstitial areas were visualized on 10–200 × magnification with Panoramic Viewer software version 1.15.2 (3DHISTECH, Budapest, Hungary).

### Statistical analysis

Statistical analyses were performed using GraphPad Prism software version 7 (GraphPad Software, San Diego, CA, USA). To test if the values were from a Gaussian distribution, Kolmogorov–Smirnov normality test was performed. Data were analyzed by two-tailed paired *t* test for all parametrical comparisons, or in the case of nonparametric data by Wilcoxon test on ranks. To test homogenization effectiveness, variances within each group were compared by Levene’s test conducted in Microsoft Office Excel (Microsoft, Redmond, WA, USA) where single factor ANOVA was applied to absolute differences of values to mean. Significance was set a priori at *P* < 0.05. Data are plotted in symbols and lines form for storability and scatter plot with mean and standard deviation for homogeneity.

## Results

### Establishment of detailed lyophilization protocols for rodent kidney, heart, liver, lung, aorta, and skin samples

Realizing the numerous possibilities offered by freeze-drying biological samples and considering the lack of definitive process parameters in the literature, appropriate protocols for various rat and mouse tissues were established.

First, a protocol for kidney samples was set up. Upon testing the same protocol proved to be applicable for several other tissues irrespective of their individual properties, such as the heart, a tissue that contains large amount of muscle fibers; the liver which is rich in lipids; and the lung, a structurally fragile tissue which collapses easier (Fig. 3a). The protocol had to be slightly modified for rat aorta and skin samples (Fig. 3b), moreover, proper pulverization was hard to accomplish due to excessive elastic connective tissue content. Results of the Karl Fischer titration showed that residual moisture content was below 5% in all investigated tissue types (Fig. 3e). Furthermore, lyophilized products were properly pulverizable and there were no signs of wet spots. Due to the smaller absolute size, same protocols can be used in the case of mouse tissues (data not shown).

### Modified protocol is applicable for non-tissue type samples

In case of human PD fluid and fecal samples primary-drying time was modified to achieve optimal cycles by prolonging the drying step and shortening the phase after it for better time management, as highlighted in the figure (Fig. 3c, d).

**Fig. 4:**
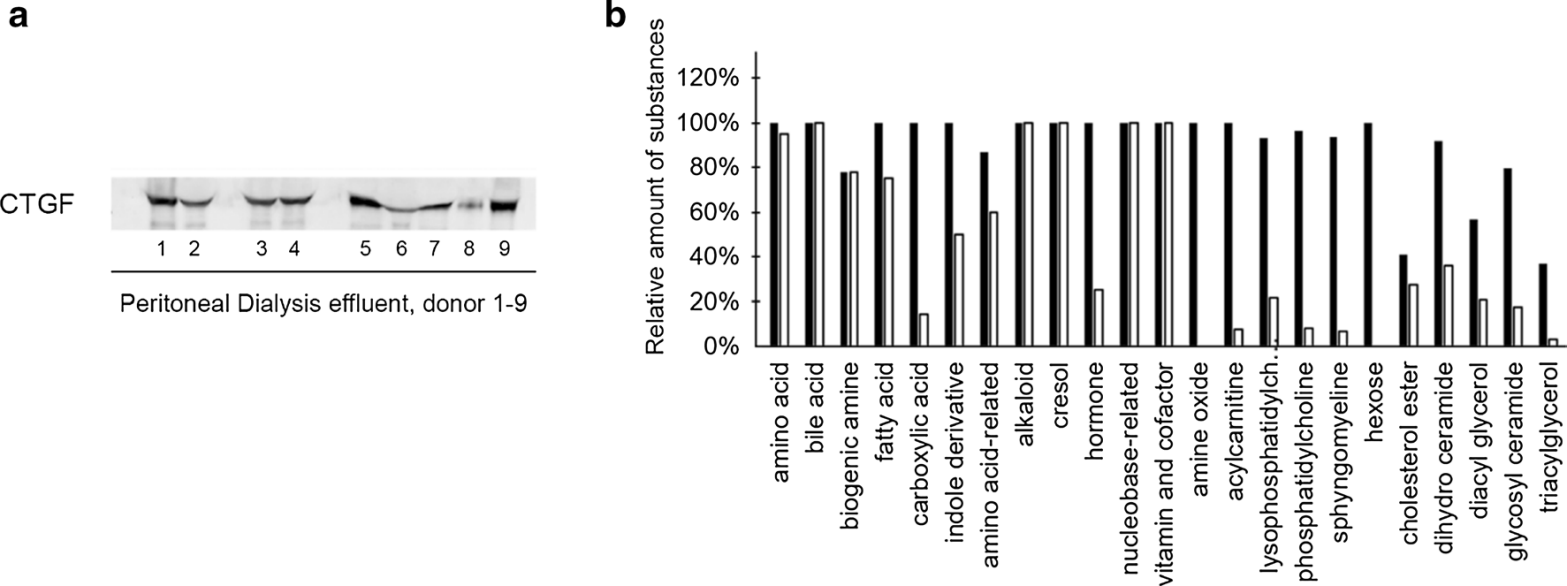
Western blot of human peritoneal dialysis effluent (PDE) and metabolome analysis of human fecal samples. **a** Representative picture for detection of connective tissue growth factor (CTGF) with Western blot from lyophilized PDE of nine human donors. **b** Relative amount of substances (*Y*-axis) belonging to various classes of compounds and detected in at least 50% of the processed feces specimens extracted with the custom protocol (filled bars) and in at least one specimen as described in Application Note 35,035 of Biocrates AG (Innsbruck, Austria, hollow bars). **b** Courtesy of Gellert Balazs Karvaly

Removing water from samples by lyophilization can also be used to concentrate solutions. We were able to establish an effective protocol, whereby × 20 concentration of PDE solution could be attained (Fig. 3c). Current research focuses on the possible use of PDE as a "liquid biopsy" to detect biomarkers of certain pathophysiological conditions. Identification of profibrotic markers such as CTGF could be a valuable method for evaluation of peritoneal fibrosis progression. However, due to their low concentration in PDE detection of these biomarker proteins is hardly possible without concentration. Detection of CTGF was successful using lyophilized PDE samples of various donors (Fig. 4a).

Water or solvent removal from fecal samples for microbiota transplantation treatment and metabolome analysis is a newly emerging concept, but comprehensive setups have not been published yet. Therefore, we set up a lyophilization protocol for human fecal samples as well (Fig. 3d). The lyophilized products have been used for metabolome analysis. The number and the relative amount of substances belonging to various classes of compounds detected in lyophilized fecal specimens was higher compared to those identified with MxP® Quant 500 KIT (method described in (Heischmann 2018)) showing the advantage of lyophilization (Fig. 4b). Of note, the Application Note also suggests consideration of freeze drying of the samples. Further data of these samples are out of the scope of the present paper and will be published in a separate article.Reference: Reference [Nail et al. (2002)] was provided in the reference list; however, this was not mentioned or cited in the manuscript. As a rule, if a citation is present in the text, then it should be present in the list. Please provide the location of where to insert the reference citation in the main body text.The reference Nail et al. 2002 remained in the reference list as a mistake. We corrected the list.

### RNA and protein stability are preserved in lyophilized samples following long-term storage

Using the above-described protocols (Fig. 3a) rat kidney and heart samples were lyophilized and stored at 4 °C to investigate RNA and protein stability during long-term storage. A few ubiquitously present proteins as well as their degradation-sensitive phosphorylated forms were detected to compare their levels in freeze-dried organ halves *vs.* their conventional frozen sample pairs after a storage-period of 20 months.

**Fig. 5:**
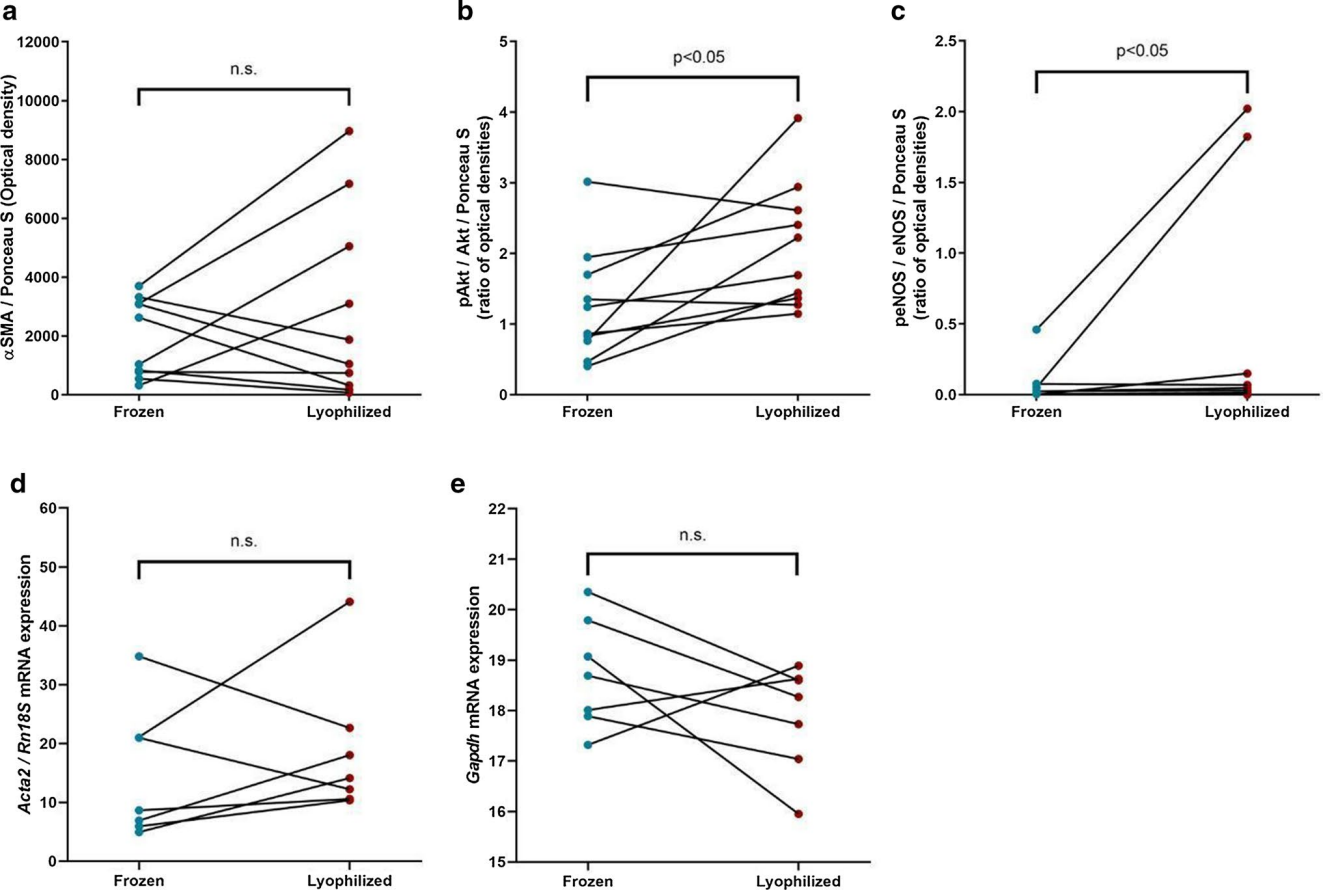
Protein and RNA stability in lyophilized *vs.* frozen sample pairs of rat kidney and heart tissues. **a** Alpha smooth muscle actin protein (αSMA) levels, **b** phosphorylated protein-kinase B (pAkt)/Akt protein ratio, **c** phosphorylation ratio of endothelial nitric-oxide synthase (eNOS) protein measured from rat kidney and heart samples with Western blot method after 20 months of storage at -80 °C in the case of frozen samples and at 4 °C in the case of lyophilized samples (*n* = 10 frozen—lyophilized sample pairs; n.s. = not significant). **d** Relative alpha smooth muscle actin mRNA (Acta2) levels and **e** absolute Glyceraldehyde 3-phosphate dehydrogenase (Gapdh) mRNA levels measured from rat kidney and heart samples with RT-qPCR method after 20 months of storage at −80 °C in the case of frozen samples and at 4 °C in the case of lyophilized samples (*n* = 7 frozen—lyophilized sample pairs, n.s. = not significant)

Kidney and heart αSMA levels were equally well detectable in both samples (Fig. 5a). Protein kinase B (Akt) and endothelial nitric-oxide synthase (eNOS) protein phosphorylation (pAkt/Akt and peNOS/eNOS ratio) levels did not decrease due to lyophilization, moreover, in case of two samples phosphorylation remained more preserved in lyophilized samples than in frozen stored ones (Fig. 5b, c). RNAs were not degraded in freeze-dried samples either; qPCR-estimated relative or absolute transcript copy numbers of Gapdh and Acta2 arbitrary target genes (Fig. 5/d, e) remained unaltered compared to conventional frozen samples.

### Lyophilization improves reproducibility

**Fig. 6:**
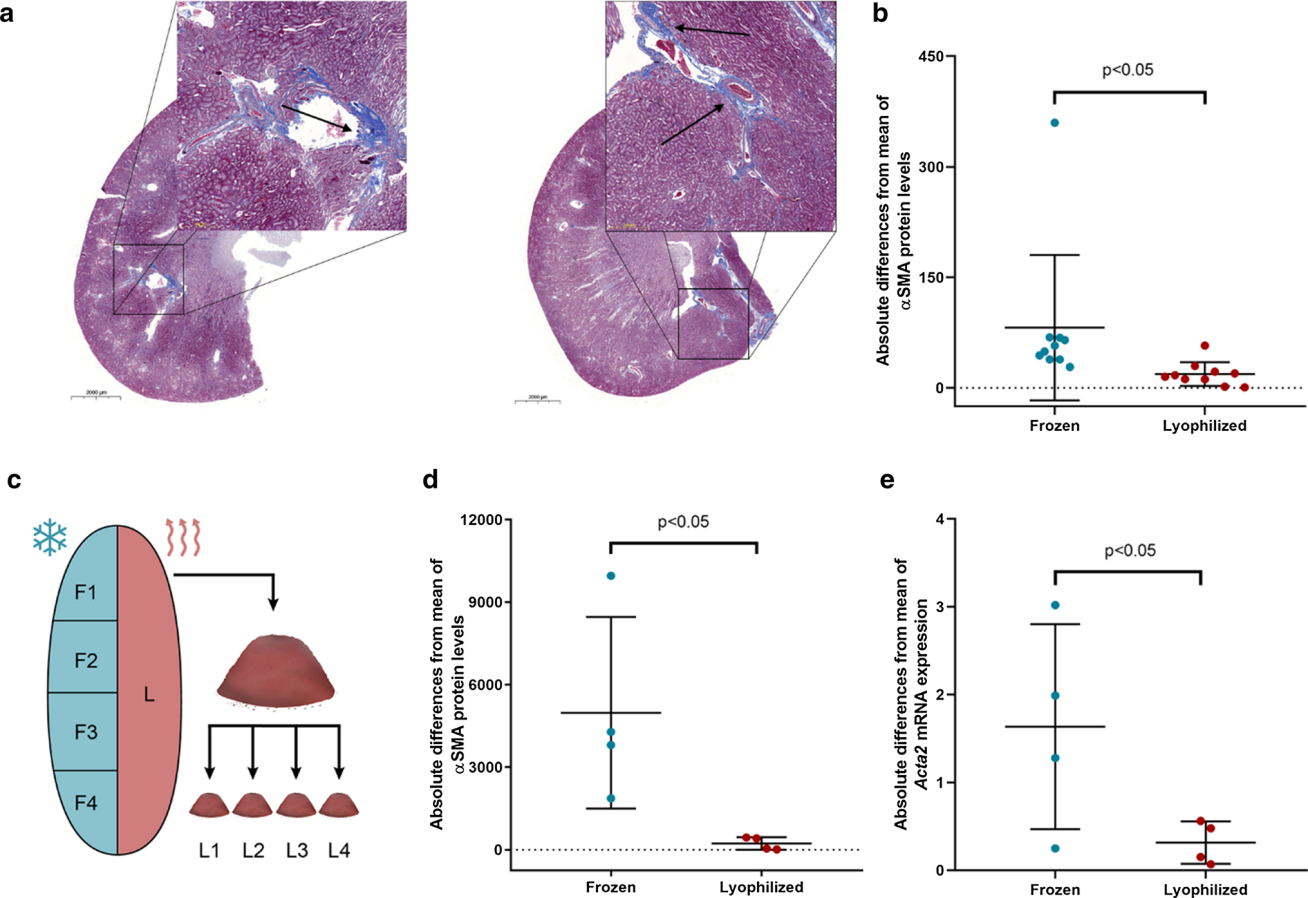
Comparison of scatter in frozen and lyophilized, pulverized samples from fibrotic kidneys. **a** Representative Masson's trichrome-stained sections of diabetic rat kidneys. Arrows show examples of focal fibrosis. (10–200 × magnification, scale bar = 2000 μm, 50 μm.) **b** Absolute differences of alpha smooth muscle actin protein (αSMA) values to mean in diabetic rat kidneys processed conventionally (Frozen) or homogenized after lyophilization (*n* = 10/group). **c** Schematic presentation of sample processing for homogeneity investigations. One half of each kidney was lyophilized (L) and pulverized, protein and RNA were isolated four times from homogenous powder (L1–L4). The other half went through conventional frozen sample processing; four pieces were cut for the isolations (F1–F4). **d** Absolute differences of αSMA protein and **e** alpha smooth muscle actin mRNA (Acta2) values to mean in frozen parts vs. lyophilized homogenous powder of the same diabetic rat kidney samples (*n* = 4 frozen parts and 4 powder portions of the same kidneys)

Fibrotic tissue accumulation is usually not homogenous, thus significant differences can develop between certain regions of the affected organ (Fig. 6a). This may cause considerable scatter and outlier data points within groups as well as between different isolations depending on the tissue region used for protein/RNA extraction (Fig. 6b). To test whether this issue can be resolved total protein and RNA were extracted from four different parts of frozen samples and 4 times from lyophilized, pulverized homogenates of the same kidney samples (Fig. 6c). αSMA protein levels and Acta2 mRNA expressions showed lower variances in the lyophilized, homogenized group than in conventionally processed frozen samples (Fig. 6d, e).

## Technical hints and possible pitfalls:

- It is better if the pump valve is open during the pre-freeze phase for faster cooling and less work for pump. On the other hand, if it stays open for lyophilization, low pressure cannot be maintained which prolongs the process and makes it less effective. Therefore, it is very important to close all valves during the drying phase.
- To achieve the most efficient heat transfer between the shelf, tray and samples, tissues can be placed directly on the tray without plastic tubes. For easier cleaning and sample labeling, aluminum foil can be used to cover the tray. Attach the foil to the tray to prevent it from flapping when releasing the pressure at the end of the process. Despite concerns about direct heat transfer possibly damaging tissue structures, we did not experience any difference either in macroscopic properties nor in storability or reproducibility compared to samples placed in tubes. We rather decided to freeze-dry samples in vials because they can be closed immediately after the lid of the instrument is opened, and chance of sample loss and contamination can be reduced.
- Release the pressure very slowly and carefully at the end of lyophilization by opening the valves gradually one by one to prevent bigger airwaves blowing away small sample pieces and spilling ice condensates onto the dry samples.
- Fecal samples can be lyophilized in their original plastic tubes with open lids.
- Liquids, such as peritoneal dialysis effluent fluids can be snap-frozen on the wall of the container by spinning in liquid nitrogen to provide larger surface for drying. The lid of the container should be open during the drying process.
- Pulverization of dried samples can be accomplished with a ceramic mortar and a pestle as well, but a TissueLyser machine with metal beads can also be used. Metal beads can be easily removed with a magnet minimizing sample loss.
- Based on our trial-and-error experiments it is highly recommended to cut solid tissue samples into tiny (~ 20 mm^3^) pieces, thus larger drying surface can be attained, which significantly increases drying efficacy. Make sure that air can reach each piece of sample.

## Discussion

Here we aimed to provide precise, detailed lyophilization protocols for animal tissues and other biological materials for laboratory research use and for clinical application as well. Our protocols are validated by product temperature measurement estimating the end of primary drying, by residual water measurement and in the case of human PDE and fecal samples by subsequent molecular biology measurements.

The graphs in Fig. 3 show temperature changes in the red frames indicate that almost all the ice was removed from the product, which means that the frozen zone turned into dried zone. Some of these protocols can be optimized further to make them more time-efficient, but they are universally suitable for different rat and mouse tissues regardless of being dense or consisting of muscle fibers (kidney, heart), having lipid accumulations (liver), being structurally fragile (lung), or containing excessive amounts of connective tissue (skin, aorta).

Residual water was determined by Karl Fischer titration, which is one of the most accurate classic analytical methods for this purpose. Values varied depending on the type of tissue, but were all below 5%, confirming efficient water removal.

PD is a successfully used renal replacement therapy in acute and chronic kidney diseases. Prolonged exposure to hyperosmotic PD fluid (high glucose content and low pH) causes functional degradation of peritoneal membrane leading to failed ultrafiltration, causing many patients to discontinue their treatment. Therefore, monitoring the function of the peritoneal membrane is crucial. Current research focuses on the possible use of PDE as a "liquid biopsy" to detect biomarkers of certain pathophysiological conditions. However, due to their low concentration in PDE detection of these proteins is hardly possible without concentration. After 20 × concentration of PDE using lyophilization we were able to detect profibrotic markers such as CTGF. Thus, lyophilization is a reliable and reproducible method to enrich low abundance but clinically relevant proteins in PDE or other biological samples.

Gut microbiota is associated with a variety of diseases and has become the subject of intensive research in recent years. Freeze drying of fecal samples has emerged as a useful tool for microbiota transplantation treatment and metabolome research (Moosmang et al. 2019; Staley et al. 2017), but comprehensive setups have not been published yet. The protocol provided here can be beneficial for laboratories utilizing high-performance liquid chromatography—mass spectrometry for metabolome analysis because removing liquid and volatile components from samples provides a highly pure, solvent free product without the degradation of components. Lyophilization of fecal samples minimizes bias caused by water content and better preserves the stability of short-chain fatty acids (Hsu et al. 2019). Furthermore, accurate water content measurement by lyophilization is also used as a reference to validate other evaluation methods such as magnetic resonance imaging (Shiguetomi-Medina et al. 2017). In the metabolome analysis, more substances belonging to various classes of compounds could be detected from lyophilized human fecal samples compared to conventionally processed specimens.Reference: Reference [Hsu et al. 2019] was mentioned in the manuscript; however, this was not included in the reference list. As a rule, all mentioned references should be present in the reference list. Please provide the reference details to be inserted in the reference list and ensure that all references are in alphabetical order.This was a replaced reference, and the reference list was not updated due to an error. We corrected the mistake, however, we could not change the order and the appropriate format of the references in the list, we kindly ask the help of the Correction Team in this matter.Reference [Nail et al. (2002)] has been replaced with [Hsu et al. (2019)] in the reference list. <p class="MsoNormal">Hsu Y, Chen C, Lin Y et al (2019) Evaluation and Optimization of Sample Handling Methods for Quantification of Short-Chain Fatty Acids in Human Fecal Samples by GC-MS. Journal of Proteome Research 18(5):1948-1957. doi: 10.1021/acs.jproteome.8b00536<o:p></o:p></p>

The protocols in this study were established on a ScanVac CoolSafe Touch Superior instrument manufactured by LaboGene A/S, Allerod, Denmark; but given the generality of exact physical parameters, highly similar steps may be applicable for other freeze dryer equipment as well (Pisano et al. 2013). General considerations and tips on how to set up a freeze-drying protocol are included in the supplementary material.

The majority of biospecimen are stored as frozen tissue at −80 °C, which can yield well preserved proteins and nucleic acids for more than a decade, however, there are several drawbacks. Frozen tissue is vulnerable to thawing (especially to repeated thawing and re-freezing, which happens often in most research labs), −80 °C freezers can generate up to 15 tons of CO_2_ yearly, are expensive (storage of a sample at −80 °C costs up to 8 times as much as a lyophilized sample), require an uninterruptible power supply and take up a lot of space. Additional issues include the need for cold chain transportation and safety concerns of liquid nitrogen tanks. A few studies demonstrated that proteins and nucleic acids are preserved in lyophilized samples in short- (Damsteegt et al. 2016; Wu et al. 2012) and mid-term storage (Mareninov et al. 2013), but to the best of our knowledge there are no data about long-term (20 month) storability.

Our results demonstrate that freeze-drying and storage at 4 °C for 20 months does not alter the quality and quantity of examined proteins or RNA molecules that can be extracted from tissue samples. Importantly, we also show that the examined post-translational modifications (phosphorylation of Akt and eNOS) are preserved and can be detected even more accurately in lyophilized samples than frozen ones.

Due to the focal characteristics of some organ injuries (e.g., fibrosis of various organs) (Schipke et al. 2017) conventional sample preparation methods might lead to false positive or false negative, hardly reproducible results in biochemical research investigations. Focality and exact localization of the injury are usually not visually predictable especially in the case of small samples obtained from rats or mice. Contrarily, freeze-dried tissue can easily be pulverized (Román et al. 2012) and a small portion of that homogenous powder used for protein or nucleic acid isolation is more representative to the whole tissue, than segmented parts from frozen samples.

This is underlined in the present study, where the focality of fibrotic tissue accumulation in the kidney is clearly demonstrated on Masson's trichrome-stained sections. Consequently, Western blot and RT-qPCR measurements of fibrotic markers from frozen samples yielded considerably higher scatter and more outlier data points compared to lyophilized, pulverized ones. Thus, we propose another advantage of lyophilization is that more consistent and more reliable results are ensured.

## Conclusions

In conclusion, this work provides a solid starting point for the utilization of freeze-drying in biological laboratory research with detailed protocols for various applications. Furthermore, it confirms that lyophilization of biological specimen preserves protein and nucleic acid integrity and can improve the reproducibility and reliability of molecular measurements. It is a cost-effective and environmentally friendly alternative to conventional frozen storage. The detailed tissue-specific protocols presented here are widely applicable and may be a valuable tool for the scientific community.

## Supplementary Information

Below is the link to the electronic supplementary material.

Supplementary file 1 (DOCX 2389 KB)

## Data Availability

Not applicable.
