# Supplementary material for: Lyophilization and homogenization of biological samples improves reproducibility and reduces standard deviation in molecular biology techniques
Source: Amino Acids. 2021 May 17;53(6):917–28. doi: 10.1007/s00726-021-02994-w (PMC8128086; doi:10.1007/s00726-021-02994-w)
Supplement: Supplementary file 1 — (DOCX 2389 KB) [file 726_2021_2994_MOESM1_ESM.docx]

Lyophilization protocol for biological samples

Instrument: ScanVac CoolSafe Freeze dryer, LaboGene

- Pre-cool the machine (set shelf temperature to -40°C) and warm up the vacuum pump (turn on the pump). Cooling is faster if you leave the pump valve open, and it is also easier on the pump with less work. Usually it takes about 3 hours to reach the desired -40°C shelf temperature (the condenser should reach -90°C, but this is achieved faster than the shelf's cooling).
- Accessory metal trays should also be pre-cooled, either in the freeze dryer, or in a -80°C freezer for faster and more effective results.

**Sample preparation**

**Solid tissues (Protocol has been set up on kidney, heart, liver, lung, skin and aorta tissues of rat and mouse)**

- - Cut the (frozen) tissue into tiny (~20 mm^3^) pieces and arrange them in 2 mL Eppendorf tubes in a way to obtain the largest possible drying surface (Sup. fig. 1/a). It is recommended to complete this step while collecting samples.
  - If necessary, pre-freeze the samples at -80°C.

**Liquid samples (Protocol has been set up on human peritoneal dialysis fluid)**

- In case of larger sample volumes (5-20 mL) it is recommended to snap freeze the liquid to the inner wall of a falcon tube by spinning/rolling it in liquid nitrogen (Sup. fig. 1/b). Thus, a larger drying surface can be obtained, which may increase the efficacy of drying.
- Fill the container to maximum 30% of the total volume.

**Sample arrangement**

- - Open the tubes and lay them on the metal trays provided with the instrument. Keep in mind not to block the openings of the tubes, and try to avoid placing them too close to the edge of the tray to avoid possible edge effect (Sup. fig. 1/c).

**
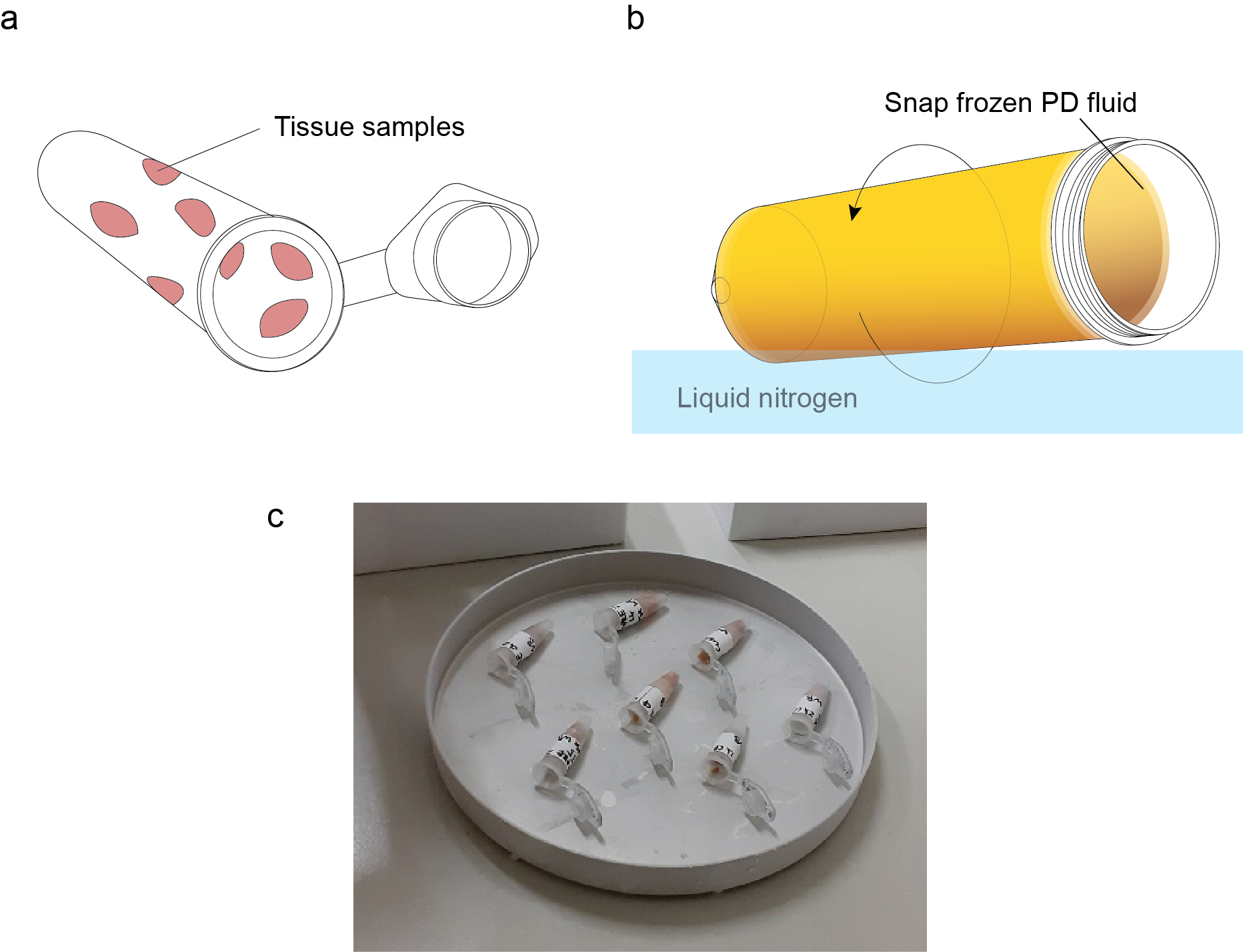
**

**Sup. fig. 1. Sample preparation and arrangement for effective freeze-drying.** Recommended preparation of a) solid and b) liquid biological samples for lyophilization. c) Optimal arrangement

- As actual shelf temperature reaches set shelf temperature carefully place the trays on the shelves. It is recommended to complete this step quickly to avoid sample and shelf warming.
- Optionally, place the Pt sensor (Sup. fig. 2) in a chosen sample piece as deep as possible. For this purpose, use a 20 G needle to prepare a hole for the sensor.

**
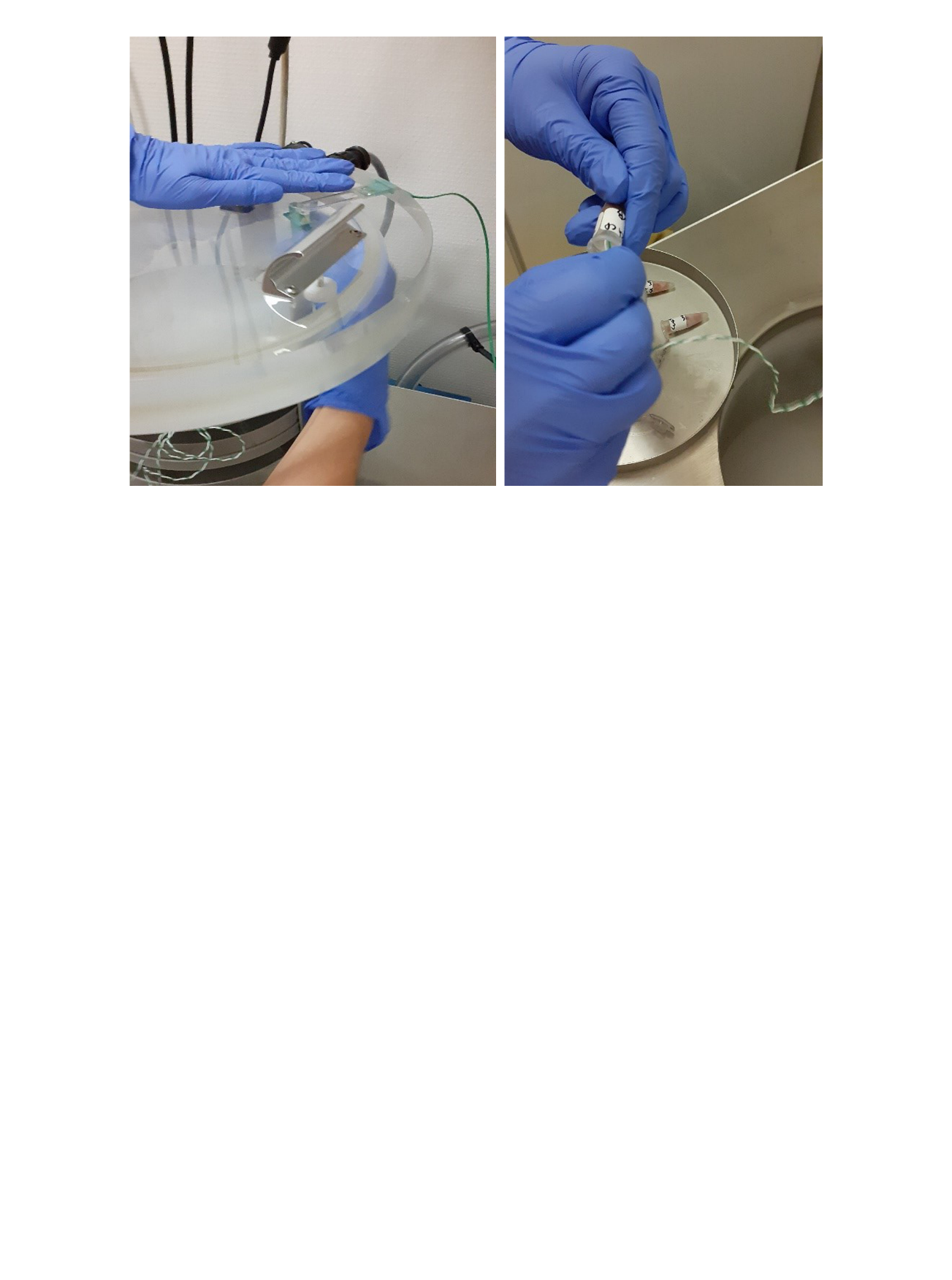
**

**Sup. fig. 2. Insertion of PT 100 temperature sensor**

**Freeze-drying:**

The freeze-drying process starts by launching a previously configured program. We provide tested protocols (Sup. table 1/a-d) for several biological samples, which may be a good starting point for optimizing freeze-drying process for specific samples and different instruments.

**Pre-freezing:**

- In general, 1-2 hours of pre-freezing should be sufficient to equalize sample (product) temperature with shelf temperature.
- During pre-freezing **vacuum pump valve** may remain open, however, it **must be closed before the start of the primary drying** step.

**Primary drying:**

- The main objective of primary drying is to sublimate ice from the sample while avoiding sample collapse. For this purpose, low pressure is applied to facilitate ice sublimation and then for faster result temperature should be raised gradually up to ~30^o^C.
- Initial chamber temperature should be set below glass transition temperature of sample components and pressure should be decreased to obtain sufficient driving force for ice sublimation on that certain temperature. For the next steps, higher pressure can be applied at higher temperature for faster sublimation.
- It takes about 10-20 hours to finish primary drying.
- If the drying proves to be incomplete, the length of the steps may be extended.

**Secondary drying:**

- In general, shelf temperature should be increased further (~40^o^C) during secondary drying.
- It takes about 3-10 hours to finish secondary drying.
- According to the general practice chamber pressure is often decreased further during secondary drying (0.1 hPa), however, there is no scientific evidence of its beneficial effect.

**End of program:**

- When the freeze-drying process is complete, first open the vacuum pump valve, then gradually open the screw on the top of the lid of the machine to slowly increase pressure and do not let the incoming air blow the samples away.
- When pressure is slightly raised by these initial steps, start the main release function of the freeze dryer machine.
- As chamber pressure reaches atmospheric pressure the machine can be opened to take out the samples, and then the de-ice program should be started to melt ice from the condenser wall.

**Sample processing:**

- While collecting samples close the lid of the tubes as fast as possible to avoid water absorption from ambient air.
- Thoroughly smash the dry samples manually with a 20 G needle.
- Add a stainless steel bead (5 mm) to each sample to pulverize them using a tissue lyser device (we used TissueLyser LT (Qiagen GmbH, Hilden, Germany). Samples are considered to be ready to use
- For storage, keep samples in a 4°C fridge. (They should remain on no higher than normal room temperature (below 25°C), so if this can be guaranteed, no cooling is needed.)
- For total RNA or protein isolation use ca. 0.4 µg powderized sample.


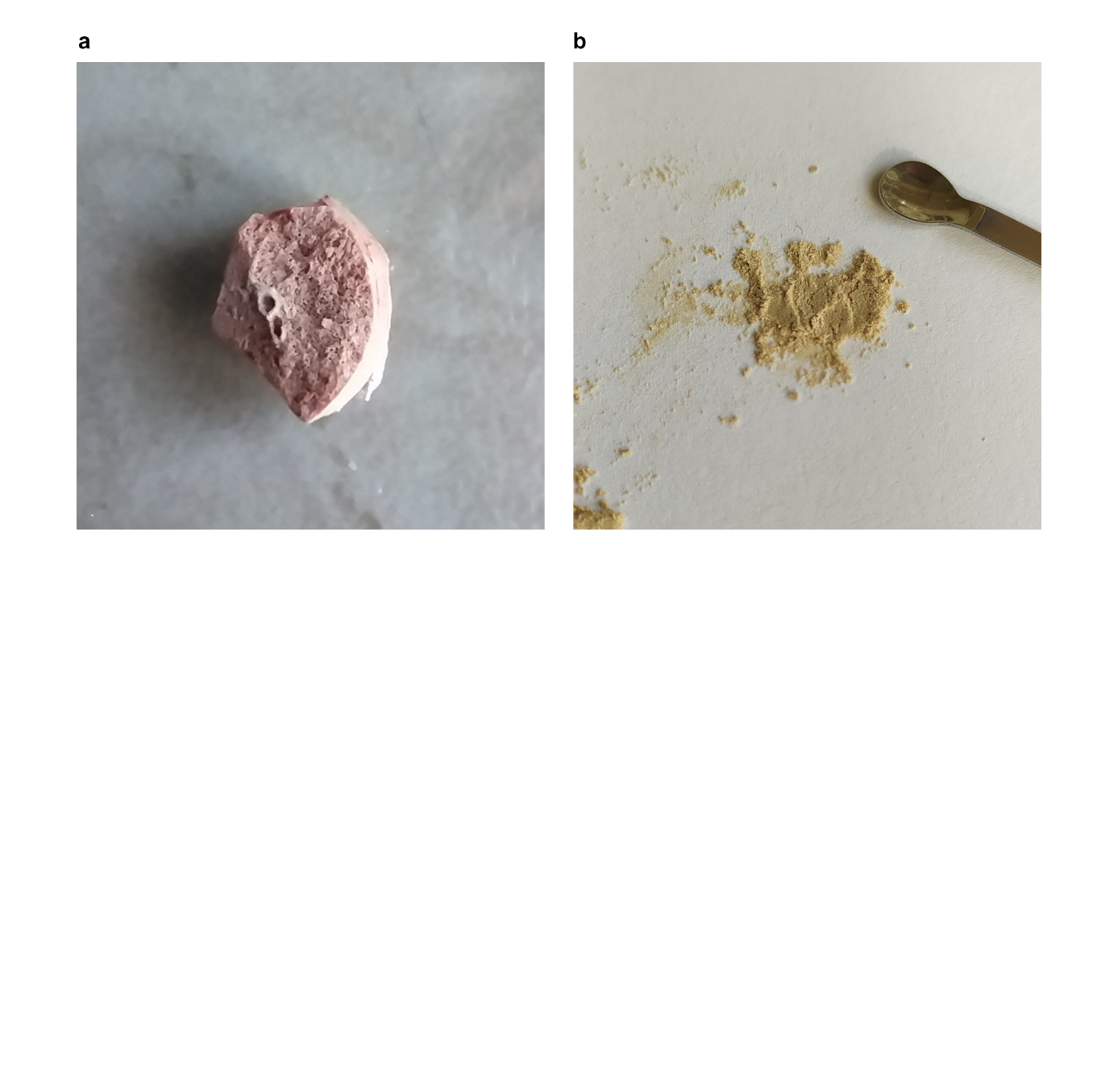


**Sup. fig. 3. Lyophilized rat lung sample a) before and b) after powderization**

| a, Kidney, heart, liver, lung | | | |  | b, Skin, aorta | | | |
| --- | --- | --- | --- | --- | --- | --- | --- | --- |
| Step | Time [h:min] | Temperature [^o^C] | Pressure [hPa] |  | Step | Time [h:min] | Temperature [^o^C] | Pressure [hPa] |
| Pre-freeze | | | |  | Pre-freeze | | | |
| 1 | 1:00 | -40 | 1013.75 |  | 1 | 1:00 | -40 | 1013.75 |
| Primary drying | | | |  | Primary drying | | | |
| 1 | 2:00 | -30 | 0.07 |  | 1 | 3:00 | -35 | 0.07 |
| 2 | 2:00 | -10 | 0.22 |  | 2 | 1:00 | -30 | 0.22 |
| 3 | 2:00 | 0 | 0.22 |  | 3 | 1:00 | -20 | 0.22 |
| 4 | 2:00 | 15 | 0.22 |  | 4 | 1:00 | 15 | 0.22 |
| 5 | 2:00 | 20 | 0.22 |  | 5 | 1:00 | 20 | 0.22 |
| 6 | 2:00 | 30 | 0.22 |  | 6 | 10:00 | 30 | 0.22 |
| Secondary drying | | | |  | Secondary drying | | | |
| 1 | 3:00 | 40 | 0.1 |  | 1 | 3:00 | 40 | 0.1 |
|  |  |  |  |  |  |  |  |  |
| c, Peritoneal dialysis fluid | | | |  | d, Feces | | | |
| Step | Time [h:min] | Temperature [^o^C] | Pressure [hPa] |  | Step | Time [h:min] | Temperature [^o^C] | Pressure [hPa] |
| Pre-freeze | | | |  | Pre-freeze | | | |
| 1 | 1:00 | -40 | 1013.75 |  | 1 | 1:00 | -40 | 1013.75 |
| Primary drying | | | |  | Primary drying | | | |
| 1 | 3:00 | -35 | 0.07 |  | 1 | 4:00 | -35 | 0.07 |
| 2 | 1:00 | -30 | 0.22 |  | 2 | 2:00 | -30 | 0.22 |
| 3 | 1:00 | -20 | 0.22 |  | 3 | 2:00 | -20 | 0.22 |
| 4 | 1:00 | 15 | 0.22 |  | 4 | 2:00 | 15 | 0.22 |
| 5 | 1:00 | 20 | 0.22 |  | 5 | 2:00 | 20 | 0.22 |
| 6 | 10:00 | 30 | 0.22 |  | 6 | 6:00 | 30 | 0.22 |
| Secondary drying | | | |  | Secondary drying | | | |
| 1 | 3:00 | 40 | 0.1 |  | 1 | 3:00 | 40 | 0.1 |

**Sup. table 1. Freeze drying programs for various biological sample types**
